# Robotic assisted laparoscopic excision of a retroperitoneal Ganglioneuroma

**DOI:** 10.1590/S1677-5538.IBJU.2016.0465

**Published:** 2017

**Authors:** Lucas Medeiros Burttet, Fernando Jahn da Silva Abreu, Gabrielle Aguiar Varaschin, Brasil Silva, Milton Berger

**Affiliations:** 1 Serviço de Urologia - Hospital de Clínicas de Porto Alegre, RS, Brasil

## Abstract

**Introduction:**

Ganglioneuromas are rare benign neoplasms of the sympathetic nervous system. We describe the case of an incidentally found ganglioneuroma in a woman. To our knowledge this is the first described case of robotic excision of a retroperitoneal ganglioneuroma.

**Case:**

A 41-year-old female had an incidental retroperitoneal mass found during a routine US. CT scan and MRI showed an 8.3cm homogeneous mass, adjacent to left kidney upper pole, with peripheral contrast enhancement. Metabolic tests were normal. Patient was positioned in a left flank position and five ports were introduced transperitoneally. A 4-arm Da Vinci SI was docked at a 45° angle to the table. Lesion was dissected along with left adrenal gland, beginning at the left renal hilum and proceeding cephalad.

**Results:**

Operating time was 325min and blood loss was 50ml. Patient was discharged after 72hours. There were no postoperative complications. Pathology showed ganglionic cells with neural tissue, and normal adrenal.

**Discussion:**

Ganglioneuromas rare benign tumors originating from neural crest and typically affect young adults. Most frequent locations are posterior mediastinum, retroperitoneum and adrenal gland. As in this case, ganglioneuromas are usually silent, slow growing tumors discovered incidentally or by mass effect. US and CT imaging may suggest the diagnosis while MRI findings can be specific for ganglioneuroma. Percutaneous biopsy is an option. Although benign, usually requires surgical excision for treatment.

**Conclusions:**

Our case shows that a robotic approach is feasible and allows for meticulous and safe dissection of vascular structures, facilitating adequate hemostasis while maintaining oncological principles.

## ARTICLE INFO

**Available at: http://www.intbrazjurol.com.br/video-section/20160465_burttet_et_al**

**Int Braz J Urol. 2017; 43 (Video #15): 997-7**

